# Anisotropic Hardening and Plastic Evolution Characterization on the Pressure-Coupled Drucker Yield Function of ZK61M Magnesium Alloy

**DOI:** 10.3390/ma17051150

**Published:** 2024-03-01

**Authors:** Jianwei You, Jiangnan Liu, Can Zhou, Wei Gao, Yuhong Yao

**Affiliations:** 1 School of Materials Science and Chemical Engineering, Xi’an Technological University, Xi’an 710021, China; liujiangnan@xpu.edu.cn (J.L.); gaowei@xatu.edu.cn (W.G.); 2 School of Mechanical Engineering, Xi’an Jiaotong University, Xi’an 710021, China; 779560229@stu.xjtu.edu.cn

**Keywords:** ZK61M magnesium alloy, hardening behavior, anisotropy, yield function, plastic evolution

## Abstract

This paper studies the plastic behavior of the ZK61M magnesium alloy through a combination method of experiments and theoretical models. Based on a dog-bone specimen under different loading directions, mechanical tests under uniaxial tension were carried out, and the hardening behavior was characterized by the Swift–Voce hardening law. The von Mises yield function and the pressure-coupled Drucker yield function were used to predict the load–displacement curves of the ZK61M magnesium alloy under various conditions, respectively, where the material parameters were calibrated by using inverse engineering. The experimental results show that the hardening behavior of the ZK61M magnesium alloy has obvious anisotropy, but the effect of the stress state is more important on the strain hardening behavior of the alloy. Compared with the von Mises yield function, the pressure-coupled Drucker yield function is more accurate when characterizing the plastic behavior and strain hardening in different stress states of shear, uniaxial tension, and plane strain tension for the ZK61M alloy.

## 1. Introduction

ZK61M magnesium alloy is extensively utilized in the domestic military industry to manufacture components that are subjected to significant mechanical stress, such as aircraft skins, panels, and interior components, as well as complex die forging parts. The incorporation of a zirconium element into magnesium alloys offers numerous benefits, for example, enhanced mechanical properties and robust overall performance. As a hexagonal dense packing (HCP) metal, the mechanical behavior of the ZK61M magnesium alloy is very complex. Selecting an appropriate yield function based on the hardening characteristics of the ZK61M magnesium alloy is advantageous in enhancing the dependability of numerical simulations for steel forming and fulfilling the requirements of real-world applications.

To study the yield behavior of metals, scholars have carried out a lot of research. The Hill48 yield criterion [1] was the first attempt to analyze anisotropy and is now one of the most widely used quadratic yield functions, providing accurate prediction of these hardening curves under uniaxial tension along different directions and under equibiaxial tension. However, due to the quadratic form of the Hill48 function, it is impossible to distinguish the difference in the yield surface of metals with different crystal structures. Hosford et al. [2,3] used an exponential form to combine the yield surfaces of body-centered cubic (BCC) and face-centered cubic (FCC) metals. Barlat et al. [4] extended isotropic functions to anisotropy by introducing anisotropic coefficients through linear transformation tensors. Barlat et al. [5,6] proposed the Yld2000-2d and Yld2004-18p yield criteria to characterize the anisotropic behavior of metals under plane and spatial stress states, respectively. Cazacu et al. [7] introduced an orthogonal anisotropic yield criterion in the form of the principal value of stress deviation to capture the anisotropy and tension–compression asymmetry. Some yield functions were established based on stress invariants. Yoon et al. [8] proposed an asymmetric yield function with the first invariant to accurately characterize the tension–compression asymmetry. Lou and Yoon [9] effectively distinguished the anisotropic behavior of metals with BCC and FCC structures by correcting for the effect of the third invariant, which was used to model the deep drawing of the 6K21 aluminum alloy [10]. Lou et al. [11] proposed a pressure-coupled Drucker yield function to simulate the strength of sheet metal between shear and plane strain, which takes into account the influence of three stress invariants: pressure sensitivity, Lode dependence, and strength difference.

In recent years, some anisotropic hardening models were proposed to capture the evolution of the yield surface through the analytical description of the anisotropy coefficient. Stoughton and Yoon [12] established the S-Y2009 yield function, and the numerical result was consistent with the hardening behavior in reality. Lee et al. [13] proposed a CQN model by the coupling of the above quadratic yield function and the Hershey–Hosford yield function. Chen et al. [14] coupled the S-Y2009 function with the Drucker function to explain the difference in the yield behavior of metals with BCC and FCC structures, which was used for the plastic evolution characterization for 304 stainless steel [15]. Hou et al. [16] proposed an anisotropic hardening model by coupling the asymmetric Hill48 function with the isotropic stress-invariant yield function. Hu et al. [17] coupled the yield criterion of fourth-order polynomials with the non-quadratic yield function under the associated flow rule to analytically describe the evolution process of anisotropic yield behavior. Wu et al. [18] established a Cazacu2004 yield function [19] with the temperature variable to describe the tension–compression asymmetry of a Mg-Gd-Y alloy under various temperatures. Lou et al. [20] proposed a stress invariant-based yield function to accurately simulate the strain hardening behavior of metals with BCC, FCC, and HCP structures under different stress states, and the convexity was analyzed by a GINCA method [21]. Hou et al. [22] proposed a constitutive model to accurately describe the anisotropy behavior of sheet metal. Zhang and Lou [23] characterized the evolving plastic behavior of BCC and FCC metals by coupling the enhanced pressure-coupled Drucker yield function and the S-Y2009 model. The yield criterion proposed by Hu et al. considers the pure shear stress along various directions to simultaneously predict the mechanical behavior under both pure shear and uniaxial tension [24]. Lou and Yoon [25] constructed an anisotropic asymmetric hardening model by coupling two Hill48 functions with Lode correlation weight functions.

Recently, Bassini et al. [26] investigated the effect of cold rolling on the microstructural and mechanical properties of a dual-phase steel. Baral et al. [27] analyzed plastic evolution and its modeling of an Al-Si-Mg die-cast alloy. Ha et al. [28] characterized the plastic anisotropy of annealed, commercially pure aluminum through experiments and modeling. Allen et al. [29] studied anisotropic hardening and texture evolution due to dislocation transmutation in twining using crystal plasticity modeling. Imandoust et al. [30] reviewed the effect of rare-earth elements on texture evolution and anisotropic hardening during the processing of magnesium alloys. Ha et al. [31] investigated the plastic anisotropy of a bake-hardening AA6013 aluminum sheet. Knysh and Korkolis [32] identified the post-necking hardening response of rate- and temperature-dependent metals. Proust et al. [33] modeled the texture, twinning, and hardening evolution during deformation of hexagonal materials. Dick and Korkolis [34] investigated the anisotropic hardening of thin-walled tubes using a new experimental method for combined tension and shear loading. Generally speaking, the more material parameters in the yield function, the more accurately the anisotropic hardening behavior can be characterized. Previous research mostly considered the anisotropic and strength differential effect, but did not consider the effect of stress states on the flow curves. The stress-state effect on the different strain hardening behaviors and their modeling is the focus of this study.

This investigation aimed to characterize the distinct hardening behavior of ZK61M in different stress states, from shear to plane strain tension, using experiments and analytical and numerical modeling. The mechanical experiments were conducted under various stress states and loading directions. The hardening behavior was analyzed by using the Swift–Voce hardening equation. The inverse engineering method was used to optimize the prediction result. A comparative analysis was conducted to assess the precision of the pressure-coupled Drucker yield function and the von Mises yield function in characterizing the plastic deformation behavior of the ZK61M magnesium alloy.

## 2. Experiment

### Materials and Experiments

**Figure 1:**
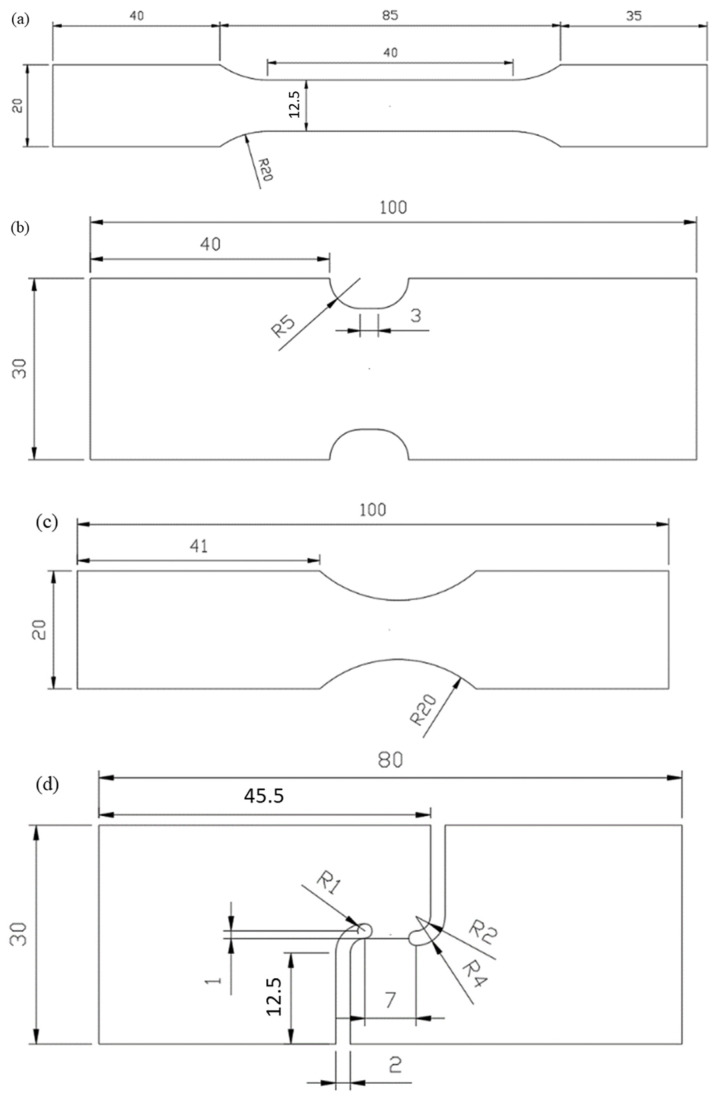
Size structure of (**a**) dog-bone specimen; (**b**) R20 notched specimen; (**c**) R5 notched specimen; and (**d**) shear specimen. (Unit: mm).

The parent material of the ZK61M magnesium alloy is a sheet metal with 2 mm thickness, which was prepared by using the rolling process. The size of these experimental specimens is shown in Figure 1, including dog bone, R20 notched, R5 notched, and shear specimens. To investigate the anisotropy of the ZK61M magnesium alloy, four specimens were cut along three different angles, 0°, 45°, and 90°, namely, RD, DD, and TD. Three replicates were conducted for each specimen in order to validate the precision and reliability of the collected data.

**Figure 2:**
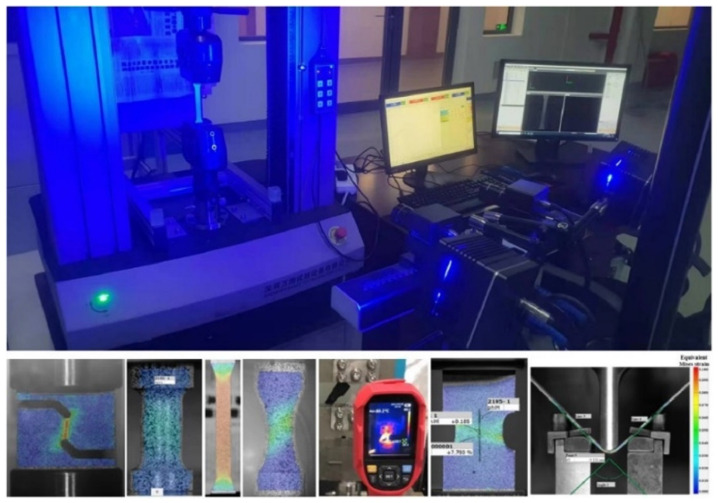
Universal mechanical experimental machine and XTOP digital image correlation system.

**Figure 3:**
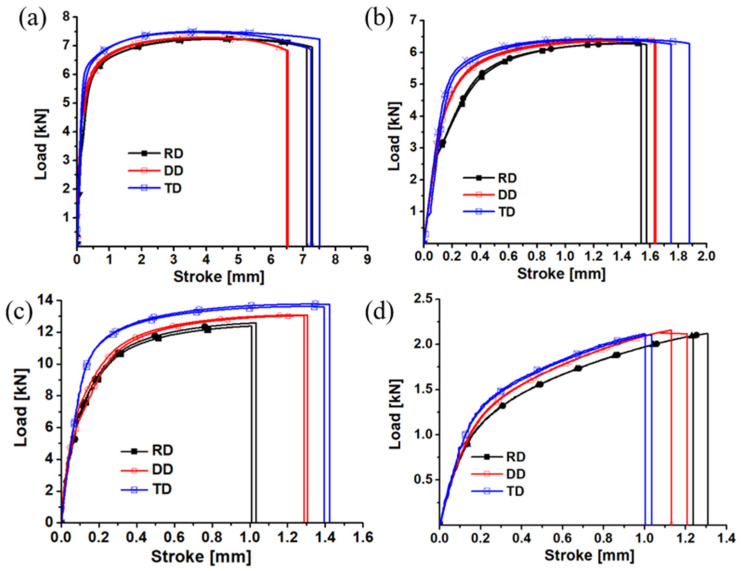
Load–displacement curves of four different samples of ZK61M: (**a**) dog-bone specimen; (**b**) R20 notched specimen; (**c**) R5 notched specimen; (**d**) shear specimen.

**Figure 4:**
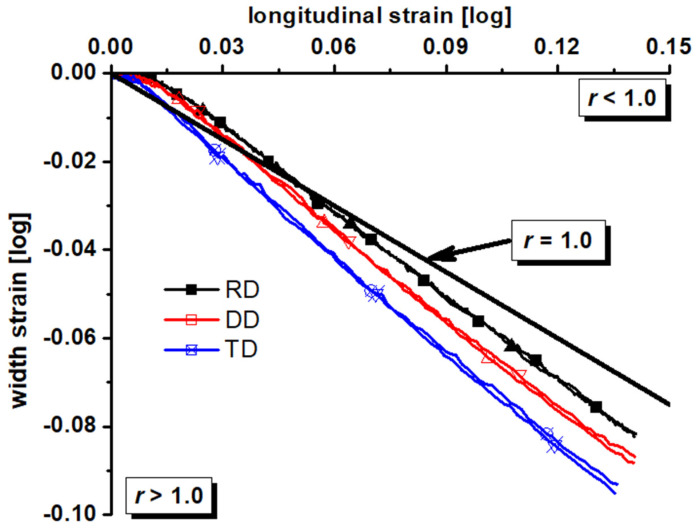
Longitudinal strain–width strain curves of the dog-bone specimen for the ZK61M magnesium alloy.

The experimental equipment is shown in Figure 2. A universal mechanical experimental machine manufactured by WANCE, Shenzhen, China and XTOP digital image correlation system in Xi’an, China were utilized to conduct tests and obtain the deformation process, respectively. The experimental apparatus is capable of preserving the symmetry and stability of clamping, hence enabling the acquisition of precise experimental load and displacement measurements. In the experimental procedure, it is important to determine the tensile velocity of these specimens. Given the quasi-static strain rate (0.001/s), the tensile velocity of the dog bone, R20 notched, R5 notched, and shear specimens was determined to be 3.6, 0.5, 0.5, and 0.5 mm/min, respectively. A 30 mm gauge was established for the dog bone and R20 notched specimens, and a 20 mm gauge was created for the R5 notched and shear specimens, as shown in Figure 1. The load–displacement curves of all tests and the longitudinal strain–width strain curves of the dog-bone specimen were obtained by the analysis of XTOP and GOM systems, as presented in Figure 3 and Figure 4.

From the load–displacement curves, the majority of the specimens have a notable degree of repeatability. It is evident that the load–displacement curve of each specimen exhibits a significant anisotropy. The dog-bone specimen exhibits a maximum strength difference of approximately 3.8%. Similarly, the R20 notched specimen demonstrates a maximum strength difference of approximately 2.2%, while the R5 notched specimen displays a maximum strength difference of approximately 9.5%. The shear specimen exhibits a maximum strength difference of approximately 3.9%. The conspicuousness of the maximum strength disparity among the four types of samples is evident. This indicates that the ZK61M magnesium alloy exhibits clear anisotropy under uniaxial tension, plane strain tension, and shear strength. From the longitudinal strain–width strain curves, the r-value also presents some anisotropy. The result indicates that the ZK61M magnesium alloy possesses the anisotropic plastic flow phenomenon.

**Figure 5:**
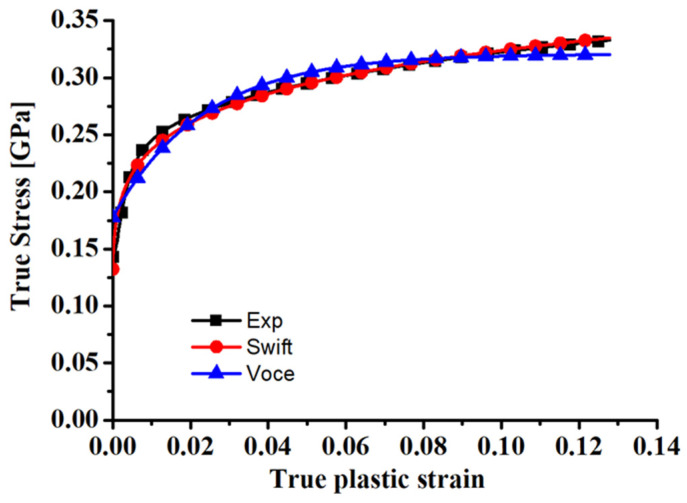
Comparison of the fitting hardening curves based on Swift and Voce hardening laws with the experimental true stress–true plastic strain curve of the dog-bone specimen along RD.

The experimental true stress–true plastic strain curves of the dog-bone specimen along RD are shown in Figure 5 and are described by the Swift and Voce hardening laws, respectively. The parameters in the Swift and Voce hardening laws are calibrated by the simplex method in the Origin 8.5.1 software. The calibrated coefficients are presented in Table 1.

## 3. Model and Method 

### 3.1. Pressure-Coupled Drucker Yield Function 

The precise modeling of sheet metal strength between shear and plane strain is of significance in ensuring the reliability of the analysis of the sheet metal forming process. In the case of nearly isotropic metals, the normalized third stress invariant exhibits equivalence between uniaxial tension and plane strain tension. It is imperative to consider the influence of pressure in order to accurately model the disparity in strength between shear and plane strain tension. Therefore, by coupling the pressure effect with the Drucker function, the following function is proposed:

(1) $$f(\sigma_{ij})=a[3b\eta +\frac{1}{3}(27-4c\xi^{2}{)}^{1/6}]{\bar{\sigma }}_{VM}$$

where ${\bar{\sigma }}_{VM}$ is the von Mises equivalent stress, η is the stress triaxiality, ξ is the normalized Lode parameter, and a, b, and c are the material parameters. Here, parameter a is calculated as

(2) $$a=\frac{1}{b+\frac{1}{3}(27-4c{)}^{1/6}}$$

**Figure 6:**
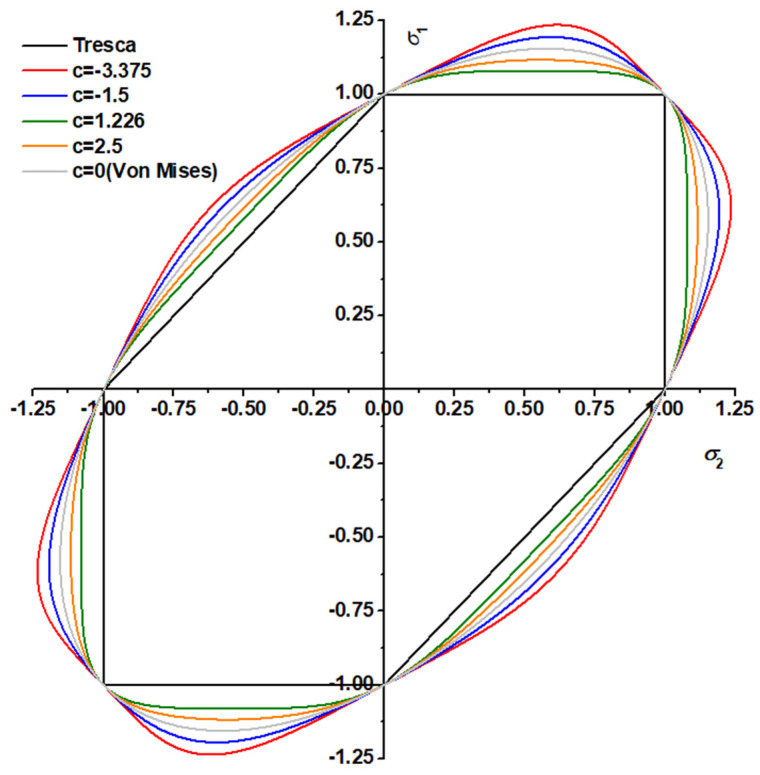
Effect of the third stress invariant on the yield surface under biaxial loading.

The value of parameter *a* depends on the stress–strain curve calculated by the experiment. Based on the conclusion that the material strength is linearly affected by the hydrostatic pressure, parameter *b* is introduced as a pressure-sensitive parameter, and parameter *c* is used to simulate the dependence of the yield on the third invariant. The value of parameter *c* ranges from −3.375 to 2.25 to guarantee the convexity of the above function, which is the same as that in the Drucker function. The effect of the third stress invariant on the yield surface is shown in Figure 6. When *c* = 0, the third stress invariant has no effect on the yield, and the yield surface is reduced to the von Mises yield surface. When *c* < 0, the Drucker yield surface around the normalized plane strain tension is greater than the von Mises yield surface, which means that higher stress is required to activate the plastic deformation around the normalized plane strain tension. On the contrary when *c* > 0, the Drucker yield surface is smaller than the von Mises yield surface, indicating that the stress required for the material to plastically deform under plane strain tension is lower. 

**Figure 7:**
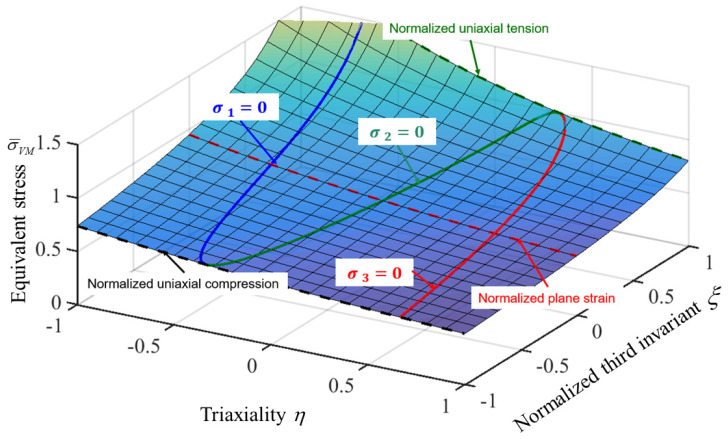
A typical yield surface of the pressure-coupled Drucker yield function with *b* = 0.05 and *c* = 2 in (η, ξ,${\;\bar{\sigma }}_{VM}$) space.

The above typical yield surface is shown in Figure 7, where the stress–strain curve is assumed to be measured by the uniaxial tensile test. Parameter *b* is equal to 0.05 to consider the pressure sensitivity, and parameter *c* is equal to 2 to couple the third stress invariant effect. Here, parameter *a* is equal to 1.6821, which is calculated from Equation (2).

The pressure sensitivity of Equation (1) enables it to model the difference between shear and plane strain tension strength, while the Lode dependence gives the yield function flexibility to characterize the different strength ratios of uniaxial and plane strain tension. Therefore, the pressure-coupled Drucker yield function can accurately predict sheet metal strength under shear, uniaxial tension, and plane strain tension.

### 3.2. Inverse Engineering

**Figure 8:**
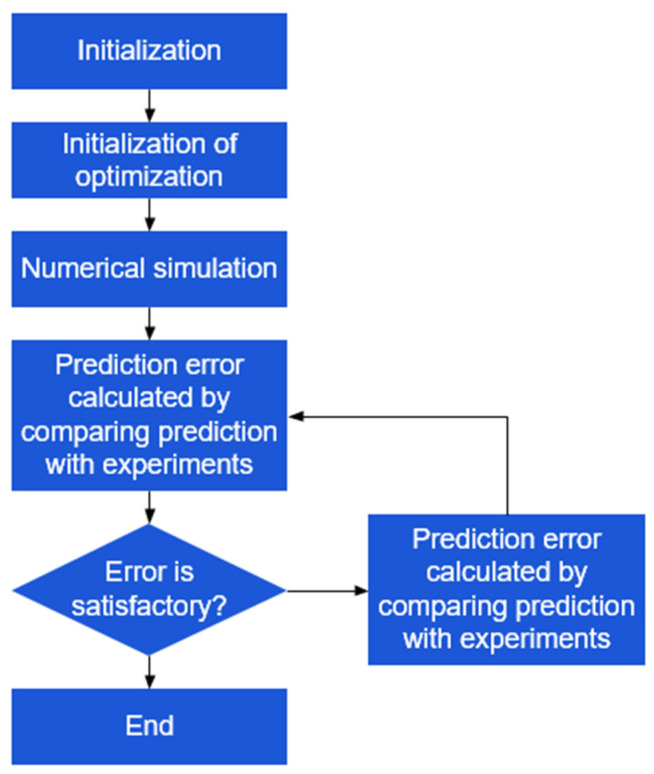
Flow chart of the inverse engineering method.

The flow chart of inverse engineering is depicted in Figure 8, which is applied to references [11,20,35,36,37]. Initially, an appropriate value is assigned as the initial value for the optimization parameter. Subsequently, the parameter is numerically simulated, and the numerical data are extracted and compared to the corresponding experimental value. The discrepancy between the numerical and the experimental values is quantified by utilizing the error function to assess the precision of the prediction. To obtain the most optimal optimization settings, it is important to minimize the error function. If the value of the error function exceeds the expected value, a simulation is conducted to predict the next optimization parameter. The updated prediction value is then compared to the experimental value once again, ensuring that the error function is less than or equal to the expected value. This process is repeated until the optimal parameter is obtained.

## 4. Comparison of the Predicted Load–Displacement Curves between Different Yield Functions 

### 4.1. Mesh Sensitivity Analysis

**Figure 9:**
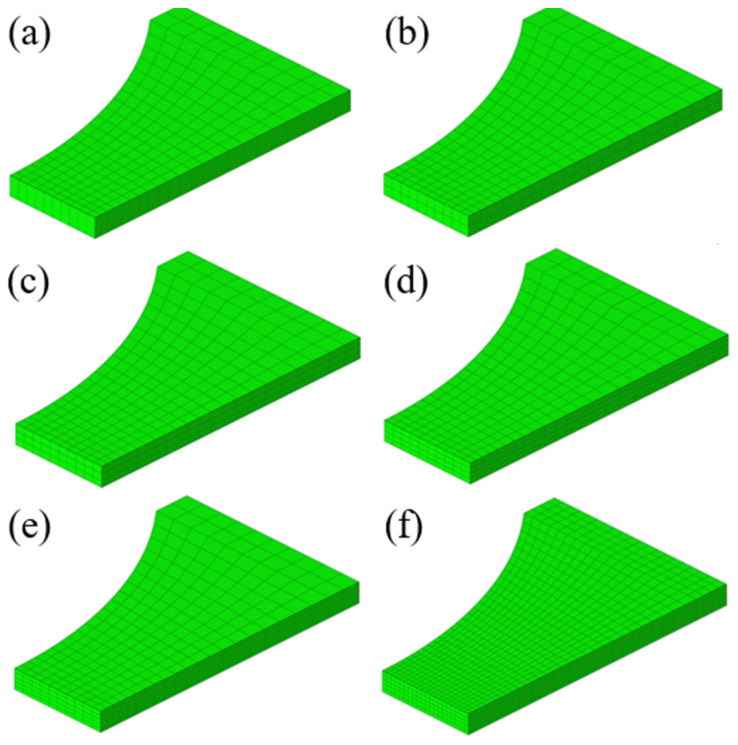
Finite element models with different mesh sizes for R20 notched specimen with different element numbers: (**a**) model #1: 220 elements; (**b**) model #2: 440 elements; (**c**) model #3: 660 elements; (**d**) model #4: 880 elements; (**e**) model #5: 1100 elements; and (**f**) model #6: 4400 elements.

The load–displacement curve of R20 notched specimens was numerically predicted by using ABAQUS/Explicit 6.14 with different element sizes to study the element size effect. The 1/8 finite element model was used to study the influence of six different element sizes on the numerical result of the R20 notched specimen, as shown in Figure 9. Three symmetric boundary constraints were applied to the numerical model accordingly because the 1/8 FE model was adopted to reduce computation time. The element used was a type of brick element with reduced integration called C3D8R. The element numbers were 220, 440, 660, 880, 1100, and 4400 for the six different meshing results in the figure, respectively. From model 1 to model 5, the number of units in the thickness direction gradually increased from one layer to five layers, and the number of units in each layer was consistent. The mesh in the thickness direction of model 6 was five layers, but the overall mesh size was smaller than that of model 5. Here, the elastic modulus in the material card was set to 45 GPa, the Poisson’s ratio was set to 0.33, and the density was set to 1.8 × 10^−6^ kg/mm^3^. The strain hardening of all models was modeled using the Swift–Voce hardening law as

(3) $$\bar{\sigma }=\frac{1}{2}[k(e_{0}+\varepsilon {)}^{n}+(A-(A-B)\exp(-C\varepsilon )]$$

where *k*, *e*_0_, *n*, *A*, *B*, and *C* are strain hardening parameters. This can be seen from the coefficients in Table 2. The flow chart of the numerical simulation is referenced in reference [38].

**Figure 10:**
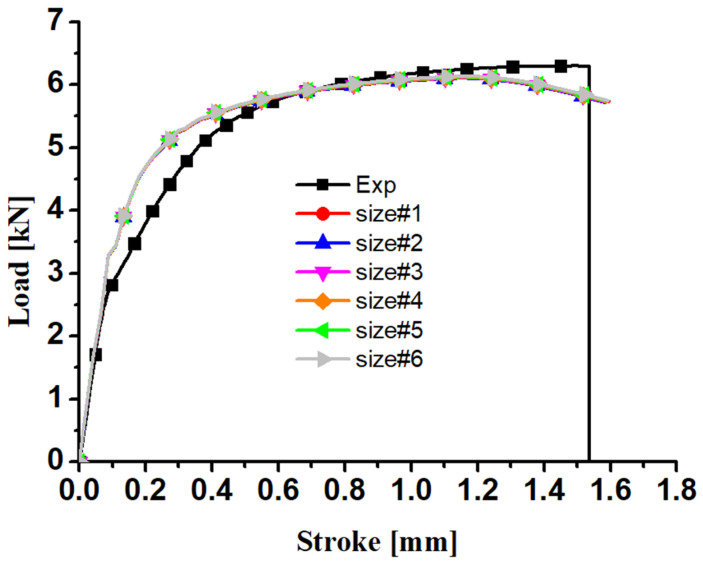
Comparison between the predicted load–displacement curves of six finite element models with different element sizes with the experimental results for the R20 notched specimen.

Figure 10 shows the predicted load–displacement curves of the six finite element models with different element sizes in Figure 9. Through a comparison with the experimental result, it is found that the element size has little influence on the numerical results of the R20 notched specimen when it is smaller than 0.5 mm in the edge length of the C3D8R elements. Therefore, numerical models with a similar element size to #1 were adopted for the simulation of all the specimens. In addition, the yield strength in the numerical simulation is different from that in the experiments, as shown in Figure 10. This is because the stress–strain curve is calibrated from the dog-bone specimens for which the stress state is uniaxial tension, while the stress state of the notched R20 specimens is between uniaxial tension and plane strain tension. The elastic regime of the numerical and experimental curves is observed to be different after the force is higher than 3.2 kN. This is because, in experiments, small plastic deformation takes place at the grain boundary, thereby reducing the slope of the force–stroke curves. The change in elastic modulus in the simulation at about 3.2 kN is expected to be caused by a numerical error in the computation of the onset of plastic deformation under small plastic deformation by the return-mapping algorithm. Last, the predicted force–stroke curve at large displacement is observed to decrease, while the experimental results do not drop. This is because the Swift–Voce hardening law calibrated by the stress–strain curve from the dog-bone specimen underestimates the strain hardening behavior under large plastic deformation. Therefore, the strain hardening behavior under large plastic strain is calibrated by the inverse engineering approach in Section 4.2 and Section 4.3 of this study.

### 4.2. Von Mises Yield Function 

**Figure 11:**
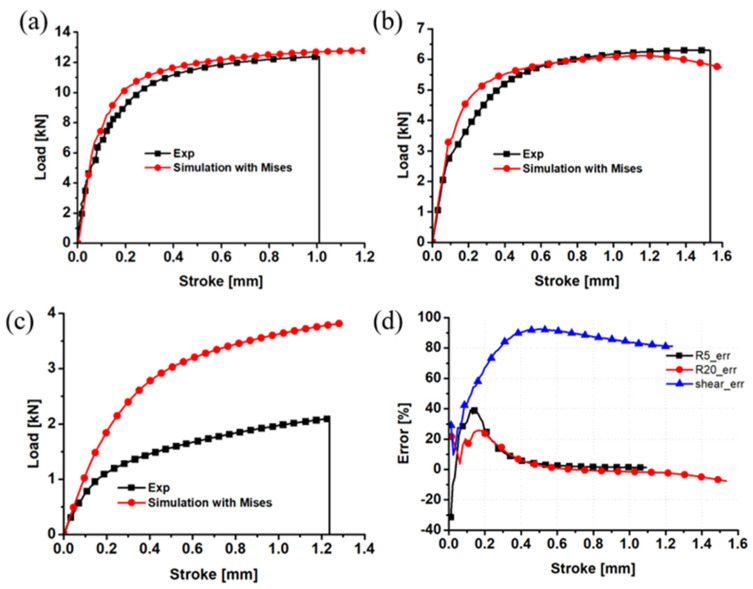
Comparison of the von Mises load–displacement curves and the experimental results for the (**a**) R5 notched specimen; (**b**) R20 notched specimen; (**c**) shear sample; and (**d**) prediction error.

The numerical simulation of R20 notched, R5 notched, and shear specimens was performed, where the yield function was the von Mises criterion. The numerical load–displacement curves were compared with the experimental results, as presented in Figure 11. Table 2 displays the parameters associated with the anticipated Swift–Voce hardening law. Clearly, the prediction error of the R20 notched specimen is approximately 20% at the elastic deformation stage, while that is approximately 5%. The error magnitude observed during the elastic stage of the R5 notched specimen is around 25%, while the error magnitude during the plastic stage is approximately 2%. The displacement increment is observed to have a significant effect on the error value of the shear specimen. Specifically, the error value initially rises, followed by a subsequent decline. It is noteworthy that the total error value is greater in magnitude. The numerical load exhibits an approximate increase of 4% compared to the experimental load of the shear specimen, and a 6% increase compared to the experimental load of the notched specimen. This indicates that the von Mises function has a tendency to overestimate the plane strain tensile strength of ZK61M by approximately 6% and the shear strength by approximately 4%. 

**Figure 12:**
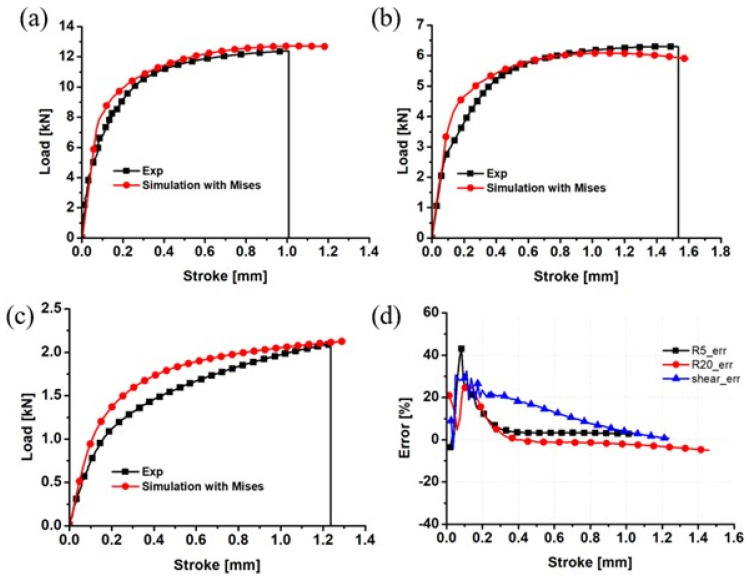
Comparison of the von Mises load–displacement curves with the inverse engineering method and the experimental results for the (**a**) R5 notched specimen; (**b**) R20 notched specimen; (**c**) shear sample; and (**d**) prediction error.

To enhance the prediction performance of the above numerical simulation, the inverse engineering method was used to optimize the Swift–Voce hardening parameters. The preliminary guess of the material parameters in Equation (3) is based on the material parameters calibrated by the true stress–true plastic strain curve of the dog-bone specimen in Table 2. The optimized material parameters are shown in Table 3. Using the optimized hardening parameters, the R20 notched, R5 notched, and shear specimens were numerically simulated, as shown in Figure 12. The comparison shows that the Swift–Voce strain hardening coefficients optimized by the inverse engineering method can more accurately characterize the strain hardening behavior of the alloy. This proves that the inverse engineering method is a promising method to characterize the strain hardening of metals under various loading conditions.

### 4.3. Pressure-Coupled Drucker Yield Function 

**Figure 13:**
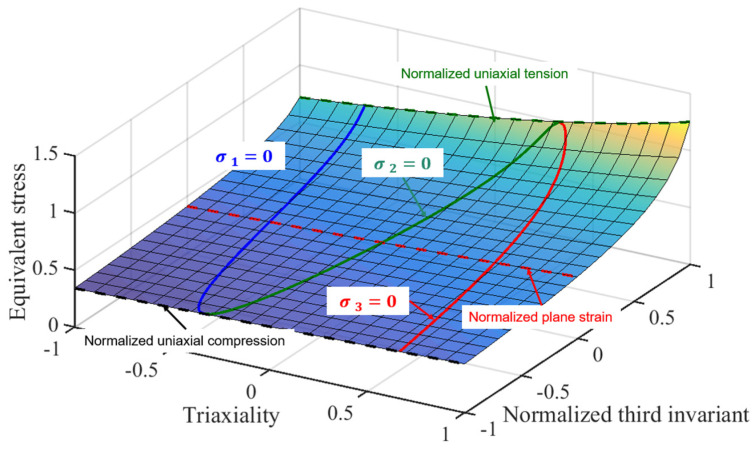
The pressure-coupled Drucker yield surface in (η, ξ, ${\bar{\sigma }}_{VM}$) space.

The evidence derived from the comparison between Figure 6 and Figure 7 demonstrates that the pressure-coupled Drucker function exhibits a satisfactory level of adaptability in the context of strength modeling for metals subjected to diverse loading situations. To ensure the precision of the numerical simulation, the pressure-coupled Drucker function was employed to estimate the load–displacement curves of material constants b and c, as well as the Swift–Voce hardening law. In order to streamline the procedure, the numerical simulation made the assumption of isotropic hardening. This decision was based on the fact that the tests conducted in this study involved loads that were approximately proportionate, and the changes in stress states were deemed to be negligible. The strain hardening model incorporates the combined model of the Swift–Voce law in Equation (3) and the hardening coefficient specified in Table 3. The inverse engineering method was employed to enhance the prediction performance of the R20 notched, R5 notched, and shear specimens. The simulation employed the associated flow rule and maintained a constant volume during plastic deformation. The optimized Swift–Voce parameters are listed in Table 4, b = −0.0268, and c = 2.2496. The yield surface in (η, ξ, ${\bar{\sigma }}_{VM}$) space is shown in Figure 13.

**Figure 14:**
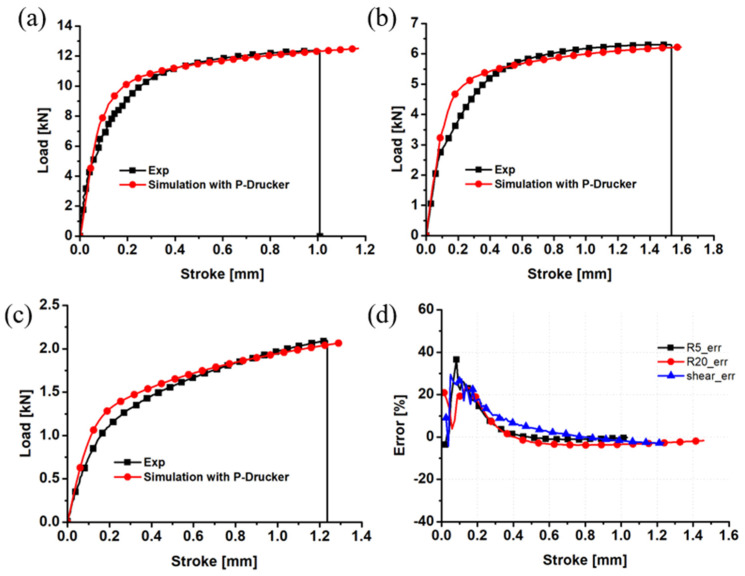
Comparison of the pressure-coupled Drucker load–displacement curves and the experimental results for the (**a**) R5 notched specimen; (**b**) R20 notched specimen; (**c**) shear sample; and (**d**) prediction error.

In Figure 14a, a comparison is made between the numerical load–displacement curve and the experimental curve. The result indicates that the pressure-coupled Drucker function accurately predicts the load–displacement curve of the R5 notched specimen prior to fracture. However, when comparing the prediction performance of the R5 notched specimen using the pressure-coupled Drucker yield function to that of the von Mises yield function, no advantage is observed.

For the prediction case of the R20 notched specimen in Figure 14b, the numerical load of the pressure-coupled Drucker function is higher than the experimental value, but it reasonably predicts the strength at the beginning of yield. After yield, the strength predicted by the pressure-coupled Drucker function is still about 3% lower than the experimental result. The pressure-coupled Drucker function can predict the trend more accurately than the von Mises function.

For the prediction case of the shear specimen in Figure 14c, the pressure-coupled Drucker function reasonably describes the trend from yield to fracture, and the predicted error value is within 3%. In addition, the maximum load predicted by the pressure-coupled Drucker function is basically consistent with the experimental value. Compared with the von Mises yield function, the pressure-coupled Drucker function was more accurate. 

## 5. Conclusions

In this paper, the plastic behavior of ZK61M is characterized from shear to plane strain tension using experiments, analytical modeling, and numerical simulation. The result shows that the plastic behavior of the metal is very complicated and cannot be properly modeled by the von Mises yield function. The pDrucker yield function can significantly improve the prediction accuracy of the load–stroke curves for shear, notched, and plane strain tension specimens. The pDrucker yield function and the combined Swift–Voce hardening law were calibrated by the inverse engineering approach and the advanced calibration approach further reduced the prediction error to less than 10% for most parts of the stroke before fracture for these three specimens. Numerical simulation showed that there is little effect of element size on the force–stroke curves predicted in numerical simulation. According to the result, the pDrucker function and the Swift–Voce hardening law are suggested to model complicated plastic behaviors of metals under wide stress states from shear to plane strain tension. The inverse engineering approach is recommended to calibrate parameters in the constitutive model to improve the prediction accuracy of numerical simulation. 

## Figures and Tables

**Table 1:** Swift and Voce hardening coefficients of the dog-bone specimen along RD for ZK61M magnesium alloy.

$\mathrm{Swift}\;\mathrm{Coefficients}\;\mathrm{in}\;\sigma_{Swift}\left(\varepsilon \right)=k{\left(e_{0}+\varepsilon \right)}^{n}$			$\mathrm{Voce}\;\mathrm{Coefficients}\;\mathrm{in}\;\sigma_{Voce}\left(\varepsilon \right)=A-{\left(A-B\right)}^{-C\varepsilon }$		
k	$e_{0}$	n	A	B	C
0.443 GPa	1.406 × 10^−4^	0.136	0.321 GPa	0.1777 GPa	43.079

**Table 2:** Swift–Voce hardening coefficients of the dog-bone specimen along RD.

$\sigma_{Swift-Voce}\left(\varepsilon \right)=\alpha \left[k{\left(e_{0}+\varepsilon \right)}^{n}\right]+\left(1-\alpha \right)[A-{\left(A-B\right)}^{-C\varepsilon }]$						
k	$e_{0}$	n	A	B	C	α
0.443	1.406 × 10^−4^	0.136	0.321	0.178	43.079	0.5

**Table 3:** Swift–Voce hardening coefficients optimized by the inverse engineering method under the von Mises yield function.

$\sigma_{Swift-Voce}\left(\varepsilon \right)=\alpha \left[k{\left(e_{0}+\varepsilon \right)}^{n}\right]+\left(1-\alpha \right)[A-{\left(A-B\right)}^{-C\varepsilon }]$						
k	$e_{0}$	n	A	B	C	α
0.443	1.406 × 10^−4^	0.136	0.321	0.178	43.079	0.584

**Table 4:** Swift–Voce hardening calibrated by the inverse engineering method under the pressure-coupled Drucker yield function.

$\sigma_{Swift-Voce}\left(\varepsilon \right)=\alpha \left[k{\left(e_{0}+\varepsilon \right)}^{n}\right]+\left(1-\alpha \right)[A-{\left(A-B\right)}^{-C\varepsilon }]$						
k	$e_{0}$	n	A	B	C	α
0.443	1.406 × 10^−4^	0.136	0.321	0.178	43.079	1.608

## Data Availability

Data are contained within the article.
